# Non-Invasive MRI and Spectroscopy of *mdx* Mice Reveal Temporal Changes in Dystrophic Muscle Imaging and in Energy Deficits

**DOI:** 10.1371/journal.pone.0112477

**Published:** 2014-11-12

**Authors:** Christopher R. Heier, Alfredo D. Guerron, Alexandru Korotcov, Stephen Lin, Heather Gordish-Dressman, Stanley Fricke, Raymond W. Sze, Eric P. Hoffman, Paul Wang, Kanneboyina Nagaraju

**Affiliations:** 1 Center for Genetic Medicine Research, Children's National Medical Center, Washington, D.C., United States of America; 2 Department of Radiology, Howard University College of Medicine, Washington, D.C., United States of America; 3 Department of Integrative Systems Biology, George Washington University School of Medicine and Health Sciences, Washington, D.C., United States of America; 4 Department of Diagnostic Imaging and Radiology, Children's National Medical Center, Washington, D.C., United States of America; 5 Department of Radiology, Children's National Medical Center, Washington, D.C., United States of America; 6 Department of Electrical Engineering, Fu Jen Catholic University, Taipei, Taiwan; Rutgers University -New Jersey Medical School, United States of America

## Abstract

In Duchenne muscular dystrophy (DMD), a genetic disruption of dystrophin protein expression results in repeated muscle injury and chronic inflammation. Magnetic resonance imaging shows promise as a surrogate outcome measure in both DMD and rehabilitation medicine that is capable of predicting clinical benefit years in advance of functional outcome measures. The *mdx* mouse reproduces the dystrophin deficiency that causes DMD and is routinely used for preclinical drug testing. There is a need to develop sensitive, non-invasive outcome measures in the *mdx* model that can be readily translatable to human clinical trials. Here we report the use of magnetic resonance imaging and spectroscopy techniques for the non-invasive monitoring of muscle damage in *mdx* mice. Using these techniques, we studied dystrophic *mdx* muscle in mice from 6 to 12 weeks of age, examining both the peak disease phase and natural recovery phase of the *mdx* disease course. T2 and fat-suppressed imaging revealed significant levels of tissue with elevated signal intensity in *mdx* hindlimb muscles at all ages; spectroscopy revealed a significant deficiency of energy metabolites in 6-week-old *mdx* mice. As the *mdx* mice progressed from the peak disease stage to the recovery stage of disease, each of these phenotypes was either eliminated or reduced, and the cross-sectional area of the *mdx* muscle was significantly increased when compared to that of wild-type mice. Histology indicates that hyper-intense MRI foci correspond to areas of dystrophic lesions containing inflammation as well as regenerating, degenerating and hypertrophied myofibers. Statistical sample size calculations provide several robust measures with the ability to detect intervention effects using small numbers of animals. These data establish a framework for further imaging or preclinical studies, and they support the development of MRI as a sensitive, non-invasive outcome measure for muscular dystrophy.

## Introduction

Duchenne muscular dystrophy (DMD) is the most common lethal genetic muscle disease diagnosed in children. Dystrophin-deficient *mdx* mice are a naturally occurring genetic model of DMD and are widely used for preclinical drug testing. Both DMD and *mdx* muscle undergo cycles of degeneration and regeneration, resulting in a chronic inflammatory state in skeletal muscle. Together, a clearly defined genetic cause and animal models establish a logical path for developing therapies for DMD through translational medicine. Several such compounds have now begun to enter clinical trials, including drug classes that target either the skipping of problematic exons [1]–[3] or inflammation and membrane stability [4].

Two significant problems encountered thus far in the case of DMD and related translational areas are a lack of quantitative surrogate outcome measures [5] and a poor success rate in translating success in preclinical mouse trials into success in human clinical trials [6]–[8]. Currently, many outcome measures used in early DMD trials consist of measures that can be subjective, could be susceptible to coaching effects or placebo effects, or show high variability [5], [9]. In preclinical *mdx* studies, most outcome measures used are unique to mice or must be substantially altered or interpreted to account for species differences.

Magnetic resonance imaging (MRI) is the gold standard for imaging damage to soft-tissue such as muscle. MRI is a non-invasive technique that does not require anesthesia in humans. It provides advantages over microCT, X-ray, and ultrasound imaging techniques in that it does not use ionizing radiation, and provides high-resolution imaging with strong contrast in soft tissues [10], [11]. Early MRI and nuclear magnetic resonance (NMR) spectroscopy studies have shown clear differences between DMD and healthy muscle. Adipose tissue replacement of muscle is prominent in standard T2-weighted MRI imaging of advanced-stage DMD patients [12], [13]. Fat-suppression MRI techniques allow for enhanced imaging of edema and inflammation [12]. Nuclear magnetic resonance spectroscopy techniques show that DMD muscle is in a state of energy deficiency [14], and detect increased lipid content within muscle [15]. Given these studies establishing dystrophic muscle phenotypes, together with studies comparing clinical groups [16], changes over time [17], and correlation with clinical assessments [18], [19], MRI is emerging as a potential key surrogate outcome measure for DMD clinical trials.

Here, we use MRI methodologies to study muscle damage and changes over time in *mdx* mice. One characteristic of the *mdx* disease is the period of peak necrosis and disease severity from 3 to 6 weeks of age; this severe disease is followed by a recovery period that produces mild phenotypes in the mice by 10–12 weeks of age [20]–[23]. We use a longitudinal strategy in which we image the same mice and muscles repeatedly from 6 to 12 weeks of age. This approach has several advantages: it examines two distinct disease phases, longitudinal measures increase statistical power, it facilitates design of non-invasive studies with technologies that are translatable to human muscle, and by assaying natural recovery periods it provides an idea of what therapeutic efficacy could look like. Here, we show clear MRI and NMR spectroscopy phenotypes in 6-week *mdx* mice in comparison to wild-type. These phenotypes include measures of muscle damage and a deficiency in energy metabolites. Interestingly, many of these differences are eliminated or reduced as *mdx* mice transition into the recovery phase of disease. Taken together, our results support the non-invasive use of MRI surrogate outcome measures for diagnosis, prognosis, and rehabilitation of muscle damage in muscular dystrophy.

## Materials and Methods

### Ethics Statement

All animal work was conducted according to relevant national and international guidelines. All studies were reviewed and approved by the Institutional Animal Care and Use Committee of Children's National Medical Center, the Washington DC Veterans Affairs Medical Center Institutional Animal Care and Use Committee, and by the Howard University Institutional Animal Care and Use Committee.

### Animal care

All experiments were conducted according to protocols approved by the Institutional Animal Care and Use Committees of Children's National Medical Center, the Washington DC Veterans Affairs Medical Center, and Howard University. Animals were maintained in a controlled mouse facility with a 12 h light: 12 h dark photoperiod, fed *ad libitum,* and monitored daily for health. All *mdx* (C57BL/10ScSn-DMD<mdx>/J) and wild-type (C57BL/10ScSnJ) female mice were obtained from the Jackson Laboratory (Bar Harbor, ME). Groups for the longitudinal study initially consisted of six wild-type and six *mdx* mice each. One wild-type mouse stopped breathing under anesthesia and was removed from the study. Mice were received at 4 weeks of age, allowed to acclimate for 2 weeks, and assayed beginning at 6 weeks of age. MRI and NMR spectroscopy were performed on each mouse every 2 weeks until the mice were 12 weeks of age. To immobilize the animals for MRI and NMR spectroscopy scans, they were anesthetized with 1.5% isoflurane, gently restrained in imaging position upon a plastic plate, and positioned in the center of the MRI scanner. For imaging, mice were placed in a holder that maintained their temperature at 37°C, with monitoring of their body temperature as well as respiratory and heart rates. Hindlimb muscles were examined in two sites per animal, including the leg and the thigh.

### MRI and NMR spectroscopy

*In vivo* monitoring of mouse hindlimbs and muscle damage was performed using a 9.4 T, 89-mm vertical bore NMR spectrometer (Bruker Biospin MRI, Billerica, MA) with a 25-mm inner diameter dual nucleus (^31^P/^1^H) birdcage coil. For anatomical positioning, a pilot image set of three orthogonal imaging planes were used. MRI pulse sequences used for data acquisition used T2-weighted imaging (T2) and fat-suppressed T2-weighted imaging (FS) sequences optimized for imaging of muscle inflammation. The imaging sequence used was a rapid acquisition with relaxation enhancement (RARE) sequence: echo time (TE)  = 7.4 ms; repetition time (TR)  = 3,000 ms; RARE factor 16; flip angle α = 90°; field of view  = 2.56 cm×2.56 cm; slice thickness  = 1 mm with no gap between slices; matrix size  = 256×256; number of averages  = 12.

Spectra were processed using TopSpin v1.5 software (Bruker Biospin MRI, Billerica, MA). For ^31^P spectroscopy studies, un-localized single-pulse spectroscopy was performed with 4k transients and a band width of 50 ppm. Integral areas of spectral peaks corresponding to inorganic phosphorous (Pi), phosphocreatine (PCr), and the three phosphate groups of adenosine triphosphate (α-ATP, β-ATP, and γ-ATP) were measured. Pi peaks were not detectable in several of the mice assayed (3 *mdx* and 5 wild-type); for these mice Pi values were uniformly omitted from the analyses. The presence of phosphomonoester (PME) or phosphodiester (PDE) peaks was also recorded; however, the signal-to-noise ratio of these peaks was not always adequate for accurate quantification. Levels of PCr and Pi were normalized by dividing by either the total ATP present in that spectrum or by the amount of β-ATP present in that spectrum. The results were consistent between both of these normalization methods; data are presented as the ratio of each parameter to total ATP.

For analysis of MRI images, two-dimensional sequential (2dseq) files were converted to digital imaging and communications in medicine (DICOM) files and analyzed using ImageJ v1.48 (NIH) software. To obtain volumetric data and account for possible variability between individual MRI slices, multiple consecutive MRI slices of each leg and thigh were assembled into image stacks encompassing 5- or 3-mm sections of the mouse hindlimb. Each stack was analyzed individually, and values for the two legs or thighs of each mouse were then averaged to obtain a single value for that mouse. For the leg, five consecutive slices along the long axis of the tibia were assayed, beginning 2 mm distal from the tibial plateau as a reference point to ensure mice were assayed at the same anatomical location. For the thigh, three consecutive slices along the long axis of the femur were assayed, beginning 6 mm proximal from the femoral condyles. Within each slice, regions of interest were digitally traced in ImageJ for each leg such that they were defined as the total area internal to the leg or thigh. Within regions of interest in each individual MRI image, we measured the total cross-sectional area, as well as the volumetric area (in voxels, or volume pixels) of bone, of muscle (with bone subtracted), and of tissue exceeding threshold signal intensity. For cross-sectional area, the highest value for each leg or thigh was recorded as the maximal cross-sectional area (CSA_max_). Bone was measured by digitally tracing the dark outline shape of the tibia or femur in MRI images, and measuring the area outlined. Muscle area was measured by subtracting bone from the combined muscle and bone area making up the full region of interest. Elevated signal intensity was measured using ImageJ software in a semi-automated manner by measuring the volumetric area in voxels that exceeded background threshold within the regions of interest. Percent of tissue with elevated signal intensity was calculated by dividing this measurement by the area in voxels measured for muscle.

### Histology

Mice were assayed by T2 imaging of the leg as described above. Immediately after each imaging session, the imaged mouse was sacrificed and the whole leg fixed in formalin. This was performed with 3 *mdx* and 2 wild-type mice at 6 months of age. Paraffin cross-sections of the legs were made at locations of interest corresponding to MRI slices as specified in a sagittal positioning image (Histoserv, Inc.), and sections were stained with H&E. Images were obtained using an Olympus BX61TRF (Olympus, Center Valley, PA) microscope with an Olympus DP71 camera and Olympus DP Controller v3.2.1.276 software. Using ImageJ software, digital tracing and overlap of the tibia structure between H&E and MRI images was used to confirm anatomical location within a corresponding MRI slice. T2 images for specific slices were then compared to corresponding H&E images for qualitative analysis within regions of interest. Multiple images taken with a 4× objective were used to produce full cross-sectional H&E montage images of the leg, and an inset image within a representative area was taken with a 10× objective. Histopathology was assayed qualitatively as reported previously [24].

### Statistical Analysis

All statistical analyses were performed by a dedicated biostatistician. Separate regression models were run for each measurement, method, and site. All models were mixed effects linear regression models with the mouse ID as the random coefficient. This approach allowed us to take into account the repeated measures taken at each time point. The main effects of strain and time were tested. All within-strain measurements were approximately normally distributed; therefore, no normalizing transformations were used. All single time-point strain comparisons were done using t-tests without adjustment for multiple testing to facilitate the powered design of trials with single time points. Sample size calculations were performed to determine the number of mice needed to detect a significant change with treatment, to facilitate future preclinical and proof-of-concept studies. Calculations were performed on *mdx* mice only, with the expectation that WT mice would not show the level of changes in inflammation and muscle changes expected in *mdx* mice. For PCr, the percent of voxels with elevated signal, maximum CSA, and volume of tissue with elevated intensity, we performed calculations to show a 20% change in mean value. Power analyses were one-sided in the direction of *mdx* value movement toward wild-type values and assumed a power of 80% and an alpha = 0.05. Throughout the text, all data are presented as means ± standard deviation unless otherwise noted.

## Results

### NMR spectroscopy shows mdx energetics deficit

**Figure 1:**
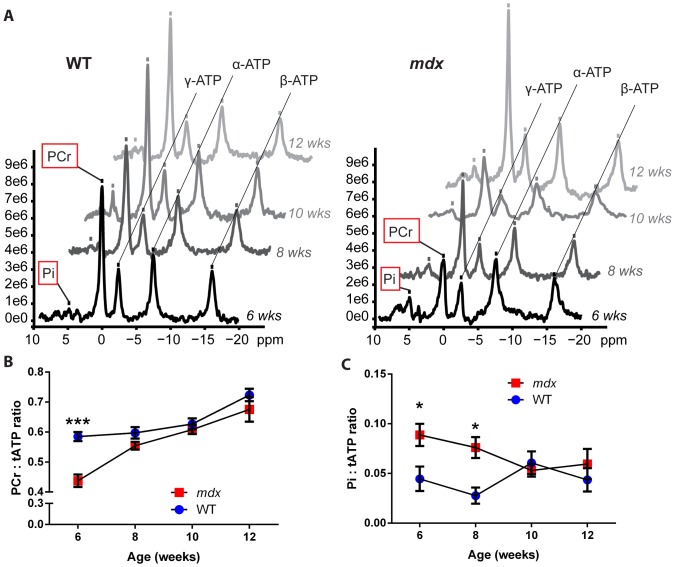
^31^P NMR spectroscopy indicates an energy deficit in 6-week-old *mdx* mice. Beginning at 6 weeks of age, *mdx* and wild-type (WT) mice were assayed by ^31^P spectroscopy every 2 weeks. A) Representative ^31^P NMR spectra illustrating the peaks of several energy metabolites in one wild-type (left) and one *mdx* (right) mouse, from weeks 6 through 12. The inorganic phosphate, phosphocreatine, and three phosphate group peaks for ATP are labeled and marked by a tick mark. Graphs are aligned by parallel lines connecting the ATP peaks; phosphocreatine and inorganic phosphate showed differences between wild-type and *mdx* mice and are highlighted by a red box. B) Phosphocreatine levels are decreased in 6-week-old *mdx* mice, then change to near wild-type levels during the *mdx* recovery phase. C) Inorganic phosphate levels are elevated at 6 and 8 weeks in *mdx* mice compared to wild-type, then change to near wild-type levels by 10 weeks. Note, peaks for inorganic phosphate were not detectable in several mice of both genotypes (3 *mdx* and 5 wild-type); values for these mice were uniformly omitted from the Pi analysis (Pi, inorganic phosphate; PCr, phosphocreatine; ATP, adenosine triphosphate; tATP, total ATP; n = 3–6 mice per data point; data are means ±SEM; *p≤0.05, ***p≤0.001).

To determine the state of energy metabolites in *mdx* versus healthy mice, we assayed the relative levels of phosphate metabolites in mice using un-localized ^31^P spectroscopy. Here, the levels of phosphocreatine (PCr), inorganic phosphate (Pi), and adenosine triphosphate (ATP, with α-, β- and γ- peaks corresponding to its three phosphate groups) were assayed every 2 weeks beginning at 6 weeks of age (Figure 1A). At 6 weeks of age, PCr levels were significantly lower in *mdx* than in wild-type mice, with PCr:ATP ratios of 0.44±0.05 and 0.58±0.03 (*p*≤0.001), respectively (Figure 1B). The difference in PCr levels between *mdx* and wild-type mice was reduced by 8 weeks of age, as the *mdx* mice entered the recovery stage of their disease, with *mdx* levels resembling wild-type and no significant difference present from 8 weeks to 12 weeks of age. In contrast to PCr, the levels of Pi:ATP were significantly increased (*p*<0.05) in *mdx* mice compared to wild-type at both 6 weeks and 8 weeks of age, after which they resembled wild-type (Figure 1C). These findings indicate that at 6 weeks of age, during the peak stage of *mdx* weakness and necrosis [20], [22], [23], the *mdx* mice experience an energy metabolism deficiency that subsequently improves during the recovery phase.

### Longitudinal MRI of mdx muscle detects effects of mdx genotype and age

**Figure 2:**
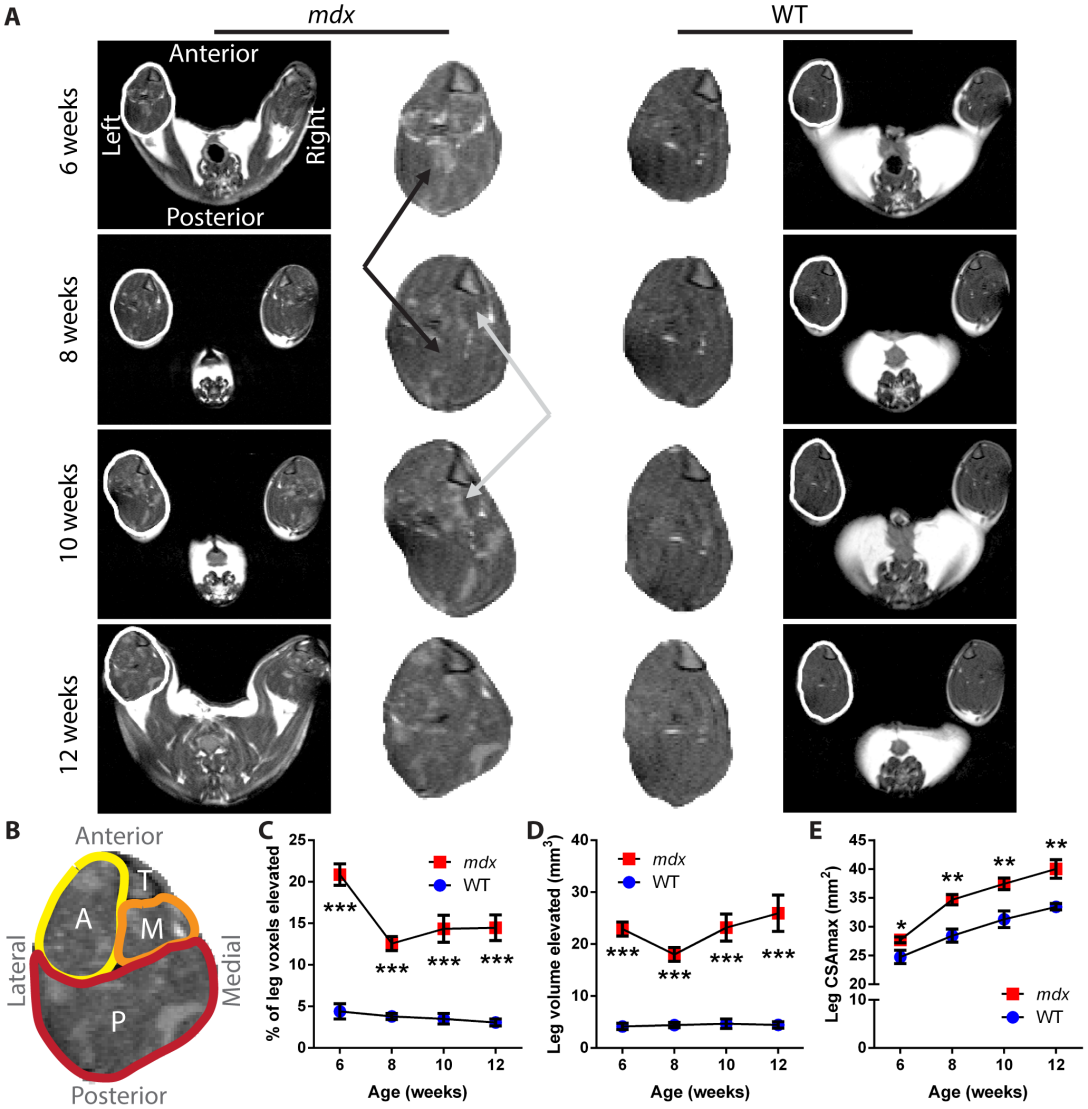
T2 of *mdx* leg shows changes in dystrophic muscle and cross-sectional area over time. A) Representative T2-weighted images from one *mdx* mouse (left) and one wild-type mouse (right) over time, each imaged from 6 to 12 weeks of age. The full MRI image of each mouse is provided on the outside column, with the leg of the left hindlimb for each mouse outlined in white and a magnified version of the leg muscles provided in the center columns. The black arrows mark a region of *mdx* muscle that showed a reduction in intensity between time points, while the gray arrows mark a region that showed an elevation of intensity between time points. The tibia, visible as a triangular structure in the upper right corner of each leg section, was used to orient the muscle slices. B) Orientation and anatomy of the leg cross sections. Anterior muscle groups (A, yellow) include tibialis anterior and extensor digitorum longus. Medial muscle groups (M, orange) include flexor hallucis and flexor digitorum. Posterior muscle groups (P, red) include gastrocnemius, soleus, and plantaris. The tibia bone is also marked (T). C) The percentage of tissue within the leg muscle that showed signal intensity elevated over the threshold that separates healthy muscle from affected tissue illustrates a change between the necrotic (6 week) and recovery phases of *mdx* disease. D) The absolute volume of tissue with elevated signal intensity detected within the leg of mice. E) The CSA_max_ values over time show the growth of muscle, and an increase for the *mdx* mice as compared to wild-type mice (n = 5 wild-type and 6 *mdx* mice; data are means ±SEM; *p<0.05, **p<0.01, ***p<0.001).

To image dystrophic muscle in live mice during the peak necrosis and recovery phases of the *mdx* condition, we performed T2-weighted imaging and fat-suppressed imaging of leg and thigh muscles every 2 weeks, from 6 to 12 weeks of age. Heterogeneous areas of elevated intensity were visible in all *mdx* mice and at all time points, in contrast to the more uniform and dark signal for healthy control muscle tissue (Figure 2A). Orientation and relevant anatomy are provided (Figure 2B). By aligning anatomically matched MRI slices using the tibial plateau as a reference point across successive time points, we observed qualitative changes in the sites and patterns of hyperintense foci within *mdx* leg muscles between two-week intervals.

Quantitatively, we detected significant effects of the *mdx* genotype on measures of muscle damage and size by both T2-weighted and fat-suppressed imaging, in both the leg and thigh muscles. No significant differences were observed in volumetric bone area between *mdx* and wild-type, for either the tibia or femur, for any of the imaging methods (Figure S1). In T2 imaging of *mdx* leg muscles, we found a significant increase in the percentage of tissue with elevated intensity (*p*<0.001) that changed over time (*p*<0.01), without a significant interaction effect being present. In T2 images of leg muscle, 6-week-old *mdx* mice had 21±3% tissue with elevated signal, as compared to 4±2% for wild-type mice (*p*<0.001; Figure 2C). As the *mdx* mice entered the recovery phase of disease, they showed a 38% reduction in the levels of affected tissue, to a mean of 13±2% of volume pixels (voxels) with elevated intensity at 8 weeks of age (*p*<0.001). These values then remained fairly steady, with no significant decrease from 8 to 12 weeks of age. These data illustrate the ability of MRI to detect significant levels of affected tissue in dystrophic legs in 6- to 12-week old *mdx* mice.

We also wanted to gain insight into whether affected muscle in *mdx* is either being eliminated or being “diluted” as the muscle grows larger and enters the recovery phase. To do this, we assayed the absolute volume of tissue with elevated intensity in the legs as well as the cross-sectional area of the legs. We detected a significant effect of the *mdx* genotype (*p*<0.001), with increased volume of elevated signal over wild-type at all ages (Figure 2D). We detected no significant effects of time on the volume of elevated signal in *mdx* mice and no significant interaction effect. Examining the sizes of muscles throughout the 6- to 12-week period assayed, we detected significant effects of the *mdx* genotype (*p*<0.001) and of time (*p*<0.001) on CSA_max_, without a significant interaction effect. Initially, the *mdx* mice showed a smaller but significant increase over wild-type in CSA_max_ (*p*<0.05), with values of 27.7±1.3 mm^2^ versus 24.7±2.6 mm^2^, respectively (Figure 2E). In contrast to the results for elevated signal, as the *mdx* mice progressed into the recovery phase, differences in CSA_max_ between the *mdx* and wild-type mice became larger, with the difference increasing by 113% from week 6 to week 8. At 8 weeks, *mdx* calves had CSA_max_ values of 34.7±2.2 mm^2^, versus 28.4±2.5 mm^2^ for wild-type (*p*<0.01), and this difference was maintained through 12 weeks of age, at which point the *mdx* mice had CSA_max_ values of 40.0±3.9 mm^2^, versus 33.5±1.3 mm^2^ for wild-type (*p*<0.01). Together, these MRI data show that as *mdx* mice recover from the necrotic phase and assume a milder phenotype [20], [22], [23], they show a decrease in the percentage of affected tissue driven by an increase in muscle size, without a complete resolution of phenotype.

**Figure 3:**
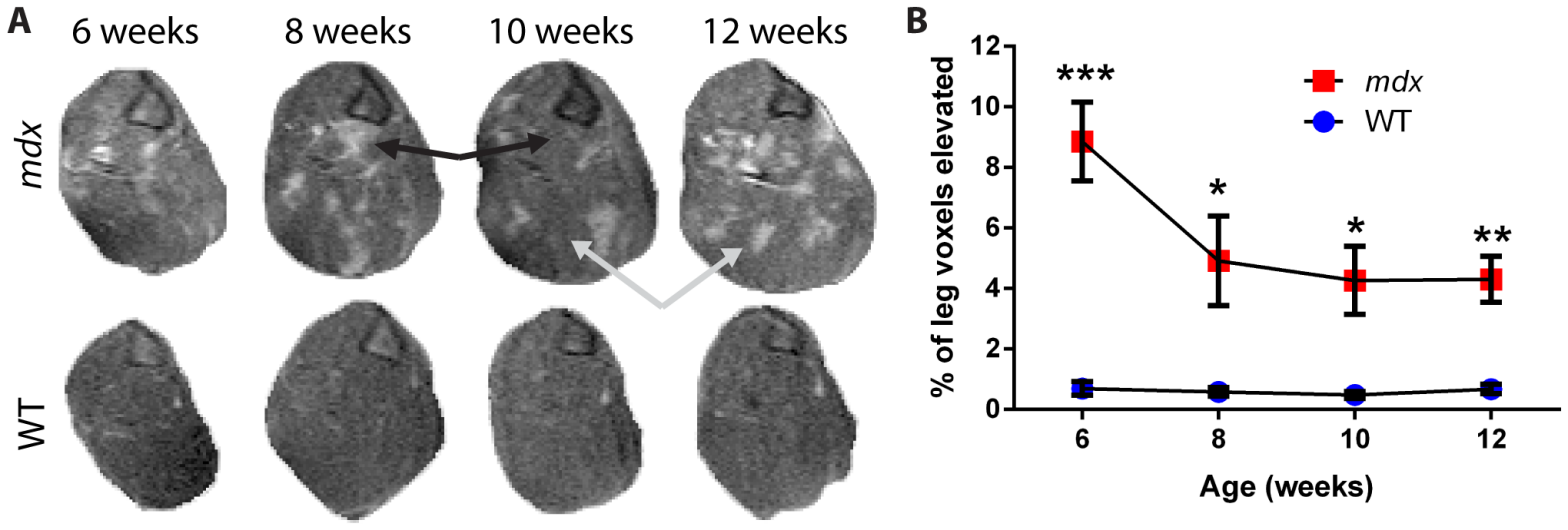
Longitudinal fat-suppressed imaging of dystrophic *mdx* leg muscles. A) Representative fat-suppressed images of leg muscle from the left hindlimb of one *mdx* (top) and one wild-type (bottom) mouse over time, each imaged from 6 to 12 weeks of age. Black arrows mark a region of muscle that showed a reduction in intensity between time points, while gray arrows mark a region that showed an increased intensity between time points. The tibia is present as a triangular structure in the upper right corner of the leg sections. B) The percentage of tissue within the leg that has an elevated signal intensity shows a difference between *mdx* and wild-type mice at all time points and illustrates a change between the peak disease (6 week) and recovery phases of *mdx* disease (n = 5 wild-type and 6 *mdx* mice; data are means ±SEM; *p<0.05, **p<0.01, ***p<0.001).

To enhance the visualization of signal from possible edema and inflammation, given the reduced signal likely from possible fatty infiltration of the muscle, we performed fat-suppressed imaging immediately following the standard T2-weighted imaging (Figure 3). Here again, we saw significant phenotypes in 6-week old *mdx* mice, with an average of 9±3% tissue with elevated intensity present in *mdx* mice, versus 1±1% for wild-type mice (*p*<0.001). Following week 6, *mdx* mice again showed a 40% reduction in affected leg tissue as they entered the recovery phase of disease, to 5±4% versus 0.6±0.3% for wild-type (*p*<0.05) at 8 weeks. Values for both genotypes were then maintained at similar levels through 12 weeks of age. These data are consistent with the T2 imaging results.

**Figure 4:**
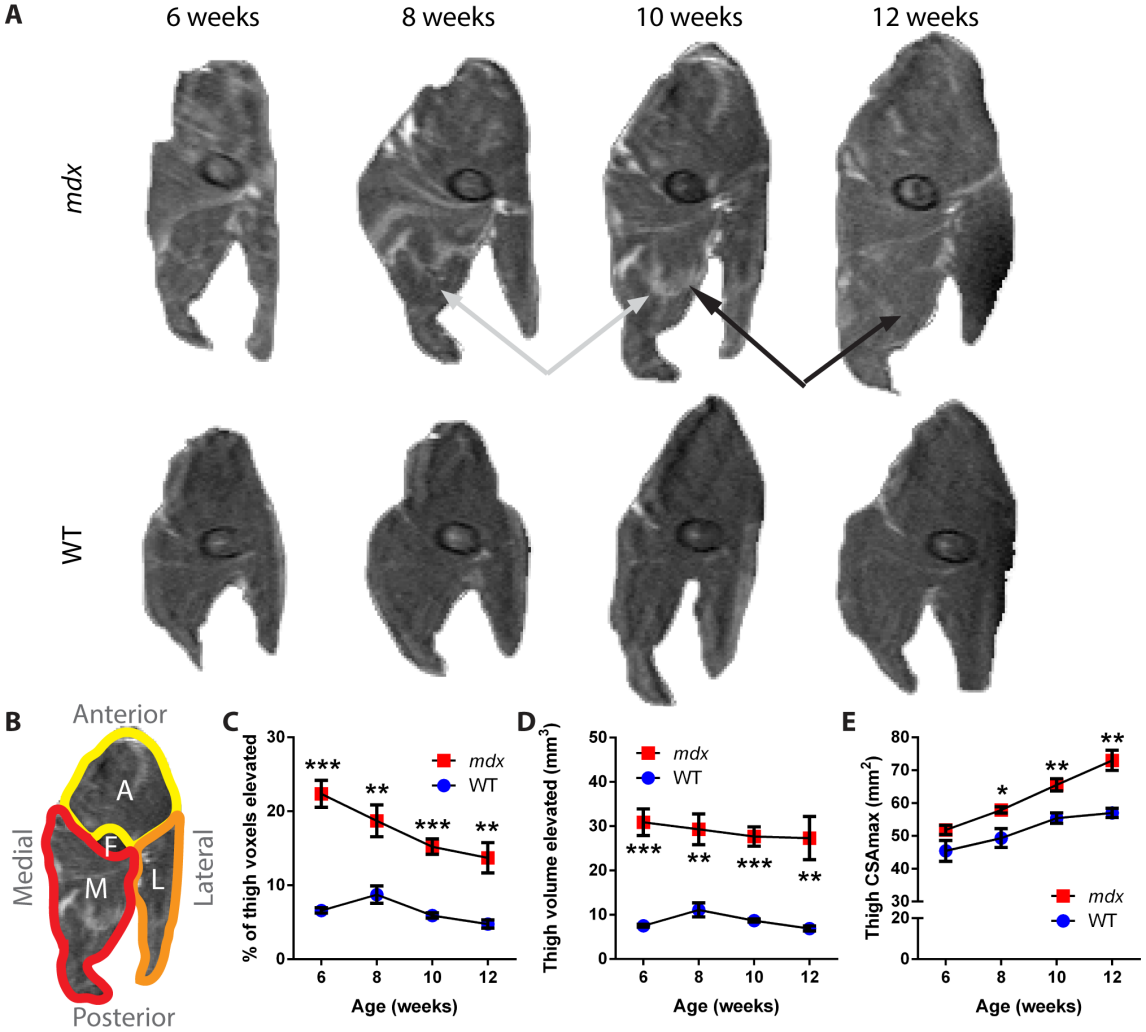
Changes in T2 imaging and cross-sectional area of dystrophic *mdx* thighs over time. A) Representative T2-weighted images of thigh muscle from the right hindlimb of one *mdx* and one wild-type mouse over the study period. The black arrows mark a region of muscle that showed a reduction in intensity over time, while the gray arrows mark a region that showed an increased intensity over time. The femur is visible as an elliptical structure towards the center of the thigh. B) Orientation and anatomy of thigh cross sections. Anterior muscle groups (A, yellow) include vastus intermedius, vastus lateralis, and rectus femoris. Lateral muscle groups (L, orange) include biceps, semitendinosus and semimembranosus muscles. Medial muscle groups (M, red) include gracilis and adductor muscles. The femur bone (F) is also marked. C) The percentage of tissue within the thigh muscle that showed a signal intensity elevated over the threshold that separates healthy muscle from affected tissue shows a difference between *mdx* and wild-type mice at all time points. D) The absolute volume of tissue with an elevated signal within the thigh of *mdx* and wild-type mice. E) CSA_max_ shows growth of the muscle size over time, and an increase in the cross-sectional area of the thigh muscle in *mdx* mice as compared to wild-type mice from 8 weeks onward (n = 5 wild-type and 6 *mdx* mice; data are means ±SEM; *p<0.05, **p<0.01, ***p<0.001).

For the thigh muscles, results were qualitatively consistent with those found for the leg muscles; we again observed changes in the patterns of affected tissue over time within the same mouse in anatomically aligned MRI slices (Figure 4A). Orientation and relevant anatomy are provided (Figure 4B). In standard T2 images, 6-week old *mdx* mice showed a significant increase in the percentage of tissue with elevated signal, with values of 22±5% versus 7±1% for wild-type mice (*p*<0.001; Figure 4C). This parameter decreased steadily over time for the *mdx* thigh, to 14±5% for *mdx* and 5±1% for wild-type mice at 12 weeks (*p*<0.01). Examining the absolute volume of tissue with increased signal independent of muscle size, we found a significant effect of the *mdx* genotype at all time points (*p*<0.01), but no significant effect of time over the ages assayed (Figure 4D). The size of the thigh muscle, as measured by CSA_max_, was not significantly different between genotypes at 6 weeks of age (Figure 4E). However, as with the leg, CSA_max_ increased for the *mdx* thighs when compared to wild-type, beginning at 8 weeks of age with values of 57.9±2.4 mm^2^ for *mdx* versus 49.3±6.4 mm^2^ for wild-type (*p*<0.05). This size differential continued to increase through 12 weeks, to 73.0±7.5 mm^2^ for *mdx* versus 57.0±3.2 mm^2^ for wild-type mice (*p*<0.01).

**Figure 5:**
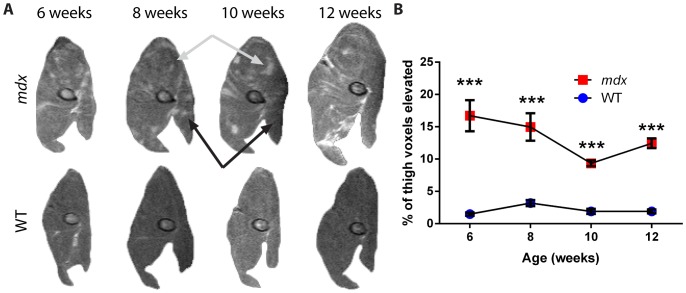
Longitudinal fat-suppressed MRI of dystrophic *mdx* thigh muscles. A) Representative fat-suppressed images of thigh muscle from the right hindlimb of one *mdx* and one wild-type mouse over the course of the study. Black arrows mark a region of muscle that showed a reduction in intensity over time, while gray arrows mark a region that showed an increase in intensity over time. The femur is visible as an elliptical structure in the central area of the thigh. B) The percentage of tissue with an elevated signal intensity within the thigh shows a difference between *mdx* and wild-type mice at all time points (n = 5 wild-type and 6 *mdx* mice; data are means ±SEM; ***p<0.001).

In fat-suppressed imaging of the thigh, *mdx* mice again showed a significant increase in the percentage of tissue with elevated signal at 6 weeks, with 17±6% as compared to 2±1% for wild-type (*p*<0.001; Figure 5). By 10 weeks of age, this decreased to 9±1% for *mdx* and 2±1% for wild-type (*p*<0.001) as the mice progressed to the recovery stage of disease. Together, thigh data are in agreement with the leg. These data illustrate that *mdx* mice show a substantial decrease in the percentage of affected tissue and an increase in muscle area as they progress from the peak disease phases [20], [22], [23] to the recovery phase of the disease.

### Histopathology present in affected areas of dystrophic mdx leg MRI

**Figure 6:**
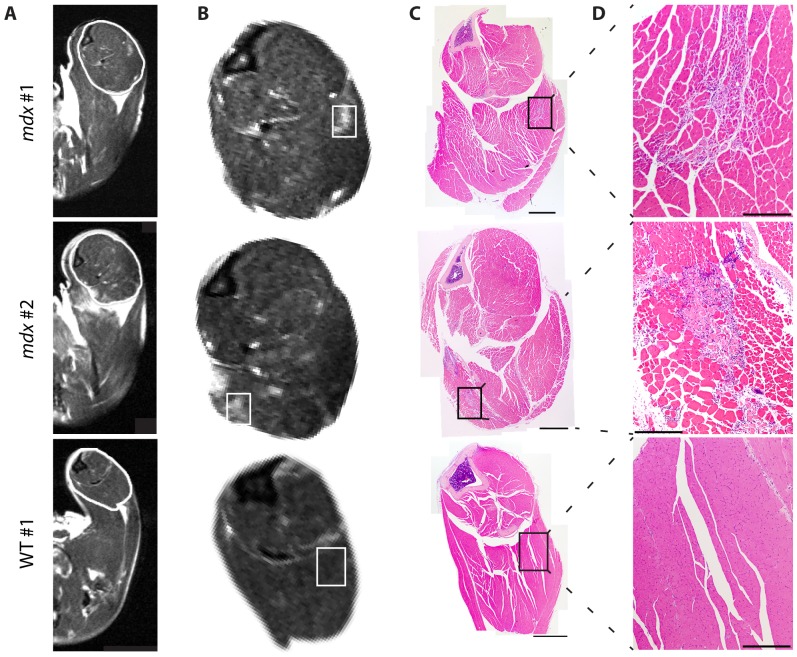
T2 imaging and histology of the *mdx* leg. Additional mice were assayed by T2 imaging at 6 months of age, followed by immediate collection of the whole leg for histology. A) Representative T2 images are provided of *mdx* (top two rows) and wild-type (bottom row) mice. The region of interest outlined in white is shown enlarged in (B). C) H&E stained cross section images corresponding to MRI slices in panels A and B. A montage image of the full leg is provided, with the inset area displayed in (D) at higher magnification (Rectangles in B and C represent the approximate areas presented in higher magnification images in D; Scale bars  = 2 mm in C and 0.5 mm in D).

To determine pathology present within areas of elevated intensity in MRI of *mdx* hindlimb muscles, we performed an additional experiment comparing H&E histology to matched MRI slices (Figure 6). Here, T2 images of the leg were obtained in 6-month old *mdx* and wild-type mice, with legs collected for histology immediately following MRI. Consistent with younger mice, *mdx* mice but not wild-type mice displayed heterogeneous patterns with foci of elevated signal intensity in their leg muscles (Figure 6A–B). By comparing matched H&E stained sections to MRI slices, we found areas of increased MRI intensity in dystrophic muscle correspond to histology regions containing a mix of inflammation, degenerating fibers, regenerating fibers, and hypertrophic fibers (Figure 6C–D). Inflammation and myofiber degeneration or regeneration were not observed in either of the wild-type controls. Results were consistent between individual mice with the same genotype. These data indicate areas of elevated intensity in dystrophic *mdx* muscle correspond to dystrophic lesions that include a combination of inflammation with degenerating and regenerating myofibers.

### Statistical power calculations suggest assays to use for lowest sample size

To determine the methodology that may be of best utility in preclinical drug or intervention efficacy studies, we performed statistical sample size calculations on MRI and spectroscopy data. Here, we calculated the sample sizes of *mdx* mice needed to detect 20% changes in *mdx* metabolite or imaging measures; in previous studies, we detect up to 40% to 50% effects from drug treatment on measures of muscle pathology and inflammation in similar aged *mdx* mice [4]. Our statistical power analyses indicated that ^31^P NMR spectroscopy performed along with T2 imaging of leg muscles at 6 weeks of age requires the lowest number of *mdx* mice to detect drug efficacy. At 6 weeks of age significant phenotypes are presented, and 20% intervention effects can be detected with 4 *mdx* mice for ^31^P phosphocreatine levels, and with 7 or 8 mice for T2 quantification of affected tissue in leg muscle (Table 1). In contrast to the leg, T2 measurements in 6-week-old *mdx* thighs required 13 or more mice for detection of drug effects. Using fat suppression acquisition methods increased the number of 6-week old mice needed for detection of intervention effects to 39 or more mice for both muscle groups. As *mdx* mice grew older, the number of mice needed to detect intervention effects in T2 images also increased to 22 or more at week 12, while ^31^P phenotypes were absent (Table S1). Together, these data indicate that a protocol of leg T2 MRI combined with ^31^P spectroscopy in mice that are 6 weeks of age provides several sensitive outcome measures for *mdx* studies.

**Table 1:** Statistical sample size calculations to detect intervention effects in *mdx* mice.

			Values at 6 weeks of age		
Method	Measure	Site	WT mean ±SD	*mdx* mean ±SD	N per group to detect a 20% change in *mdx*
^31^P NMR Spec	PCr: tATP		0.585±0.030	0.438±0.047	4
T2	% elevated signal	Leg	4±2	21±3	8
	Vol. elevated (mm^3^)	Leg	4.16±1.36	22.91±3.31	7
	% elevated signal	Thigh	7±1	22±5	13
	Vol. elevated (mm^3^)	Thigh	7.52±0.75	30.89±7.33	18
Fat Suppression	% elevated signal	Leg	1±1	9±3	41
	Vol. elevated (mm^3^)	Leg	0.70±0.49	10.00±3.55	39
	% elevated signal	Thigh	1±1	17±6	39
	Vol. elevated (mm^3^)	Thigh	1.69±0.73	23.34±9.04	58

*Abbreviations:* NMR Spec, Nuclear Magnetic Resonance spectroscopy; PCr, phosphocreatine; tATP, total adenosine triphosphate; Vol., Volume; WT, wild-type.

## Discussion

MRI shows promise as a surrogate outcome measure for DMD that is capable of non-invasively detecting muscle damage in patients. Here we use magnetic resonance technologies to identify and longitudinally characterize phenotypes in the *mdx* model of DMD. Since *mdx* mice naturally show a peak of necrosis, weakness and disease at 3 to 6 weeks of age [20]–[23], followed by a natural recovery phase in which they show only mild skeletal muscle disease, the peak disease phase is commonly used to assess preclinical efficacy of therapeutics [4], [25]. We find *mdx* mice show significant imaging and spectroscopic alterations during this peak disease phase. Furthermore, these changes decrease as mice progress to the recovery phase. Our findings indicate non-invasive MRI and NMR spectroscopy are sensitive outcome measures that can be used to study disease and evaluate potential therapies in the *mdx* model of muscular dystrophy.

Energy metabolites detected using ^31^P spectroscopy show a significant deficit of phosphocreatine in 6- to 8-week old *mdx* mice. Significant deficits in phosphocreatine and increased inorganic phosphate are also found in DMD patients [14]. Since energy for muscle contractions comes from phosphocreatine, which is used for generation of ATP through a reversible reaction with creatine phosphokinase, the PCr:ATP ratio is reflective of the energy state of muscle [26]–[28]. Thus, the decrease in PCr:ATP reflects a muscle bioenergetics deficit in both dystrophic 3- to 12-year-old DMD patients [14] and 6-week-old *mdx* mice. Similar results have been found in *ex vivo* cardiac studies of *mdx* mice, where a decrease in PCr is found in association with a decrease in mitochondrial content of heart tissue [29]. Consistent with heart muscle, we and others find significant mitochondrial deficits in *mdx* skeletal muscle [30]. Other muscle disorders such as mitochondrial myopathies and polio paralysis show a deficit in phosphocreatine levels as well [31], [32]. Interestingly, we find the PCr:ATP ratio in *mdx* increases to a level not significantly different from wild-type by 8 to 10 weeks of age. This illustrates an improvement in energetics of dystrophic *mdx* skeletal muscle during the period associated with recovery.

MRI of *mdx* muscle provides significant phenotypes at all ages examined, characterized by hyper-intense foci and a more heterogeneous appearance. Histology shows these imaging phenotypes correspond to dystrophic lesions containing a mix of inflammation with degenerating, regenerating, and hypertrophic myofibers. This is consistent with Walter et al, who find hyperintense regions are consistent with dystrophic lesions and damaged myofibers enhanced by contrast agents, and who use ^1^H spectroscopy in *mdx* to show minimal fatty infiltration in comparison to DMD [33]. We see foci of hyper-intense signal change over time, consistent with a dynamic disease process [20] and with time frames established for muscle repair following crush injury [34]. We find cross-sectional area of *mdx* muscle increases over time, while absolute volume of dystrophic lesions in imaging does not. Data in the literature indicate such increases in CSA_max_ are the result of hypertrophy and regeneration [23], [35]–[37].

Comparing spectroscopy and imaging results, there is a discrepancy in *mdx* mice. Spectroscopy shows an initial energetics deficit that is eliminated by 8–10 weeks, while imaging phenotypes improve but persist at all ages examined (including 6 months). Established muscle histology and function data may provide insight into these differences. Through matched histology, we find *mdx* imaging phenotypes are consistent with sites containing inflammation along with myofiber degeneration and regeneration. Previous studies establish this histopathology peaks at 3–6 weeks, then improves but persists throughout the *mdx* lifespan [20]–[23]. In a longitudinal study we are unable to assay isolated muscles for function, but isometric force data in the literature establish muscle function at stages we examined. Throughout the lifespan of their disease, *mdx* muscles show deficits in normalized strength, where force is measured relative to mass (kN/kg) or cross-sectional area (kN/m^2^) [4], [23], [25], [37], [38]. However, raw absolute force measurements (expressed in kN or mN) behave differently. During peak *mdx* disease (within ages 3–7 weeks), *mdx* muscles show deficits in absolute tetanic forces for extensor digitorum longus (EDL) [23], soleus [23], tibialis anterior (TA) [21], and diaphragm [39]. As *mdx* enter a recovery phase (approximately 2–8 months of age) strength deficits improve [20] and raw measures of isolated EDL [23], [37], soleus [37], and TA [38] muscle forces are usually at or above wild-type levels. Comparing these observations and stages, it may be possible that energetics deficits play a role in decreased raw isometric forces during peak *mdx* disease. Consistent with this, creatine treatment targeting energetics deficits in DMD is found to both increase phosphocreatine and preserve muscle function over placebo in a short term study [14]. Alternatively, there may be a threshold of inflammation and muscle damage that manifests as metabolite or force deficits, and mice may cross this during recovery. More investigations will be needed to clarify the relationship of energetics, histopathology, and strength in *mdx* muscle.

Though some measures are consistent between *mdx* and DMD, their disease courses have clear differences. A main difference is that DMD is progressive. As children age they show increasing weakness, fibrosis, and infiltration of muscle with fatty tissue. MRI and NMR studies of DMD (summarized in Table 2) show striking differences from controls as fatty adipose tissue replaces muscle [13], [15], [40], [41]. In DMD, edema is observed within damaged muscle [12]. At advanced ages, *mdx* disease does eventually progress, with injury phenotypes becoming more pronounced after 8 months [38], cardiac deficits around 9 months [42], [43], and advanced histopathology with susceptibility to rhabdomyosarcoma around 2 years [44]. However, *mdx* typically do not progress to a point with the degree of muscle wasting and fatty infiltration apparent in DMD. The *mdx* stages we examine here may be most consistent with early DMD, where muscle shows weakness, pathology and inflammation, but patients do not yet exhibit extensive replacement of muscle with fibrotic and fatty tissue. Moving forward, many gene therapy, antisense oligos, and next-generation drug strategies will indeed want to target early DMD stages to prevent muscle loss and to target stages with more myofibers present.

**Table 2:** MR imaging and spectroscopy phenotypes in dystrophinopathies.

Reference	Study Description	Assay	Study population	Findings in dystrophy	Our findings in *mdx*
*Banerjee * [*57*]	DMD vs. controls; effects of creatine	^31^P NMR Spectroscopy	DMD; 27 patients, 8 controls	**PCr is lower & Pi is higher** in DMD	PCr is lower and Pi higher in 6 week *mdx*
*Forbes * [*58*]	Ambulant DMD vs. controls	T2 MR Imaging	DMD; 30 patients, 10 controls	**CSAmax higher** in DMD (MG, Sol, ST)	CSAmax up for *mdx* in all weeks (leg & thigh)
*Kinali * [*13*]	Leg muscle of DMD	T2 MR Imaging	DMD; 34 patients	**Non-muscle content** and fat higher in DMD	Non-muscle higher in *mdx* muscle in all weeks
*Newman * [*15*]	Forearms of DMD vs. controls	^1^H NMR Spectroscopy	DMD; 6 patients aged 9–15 years	Fat content higher in DMD	No fatty infiltration visible in *mdx*
*Kim * [*12*]	T1 and FS imaging of DMD pelvic muscles	Fat-Suppressed T2 Imaging	DMD; 42 patients	DMD Edema; GMa, VL, GMe most frequent	Inflammation and muscle damage present in *mdx*
*Dunn * [*53*]	Quantitative MRI of *mdx* vs. WT	T2 Mapping	*mdx*; 32–48 weeks	T2 decrease, ^1^H density & water increase	Inflammation and muscle damage present in *mdx*
*Zhang * [*29*]	Cardiac function and metabolism in *mdx*	MRI & *ex vivo* ^31^P NMR Spec	*mdx* and WT; 32 weeks	**Decreased PCr in heart**; RV & LV defects	Decreased PCr in skeletal muscle
*McIntosh * [*34*]	Crush injury and *mdx* vs. controls	T2 images	*mdx*; 8–10 weeks	Dystrophic foci seen; **muscle changes over 21 days** post-injury	Changes in natural *mdx* lesions between 2 to 4 week intervals
*Stuckey * [*45*]	Cardiac morphology and function in *mdx* vs. controls	Longitudinal cardiac & Gd MRI	*mdx*; 4–52 weeks	RV Dysfunction by 1 & LV by 12 months; fibrosis by 6 months	Heart fibrosis after 6 months; 8 weeks if dosed with prednisone[4]
*Pratt * [*55*]	Case study of a single *mdx* leg	MRI	One single *mdx* mouse; 5–80 weeks	Peak in MRI hetero-geneity, recovery after 13 weeks	Peak phenotypes in necrotic phase, damage persists at 8–12 weeks
*Straub * [*46*]	Agent-enhanced MRI of *mdx* and *Sgca^−/−^* mice	MS-325 agent MRI	*mdx* & *Sgca^−/−^*; 8–10 weeks	Enhances dystrophic muscle contrast	
*Amthor * [*47*]	Albumin targeting of dystrophic muscle	Gd enhanced MRI	*mdx*; 11–13 weeks	HSA targets to dystrophic muscle	
*Odintsov * [*48*]	MRI detection of transplanted stem cells	MRI of labeled stem cells	*mdx* and dKO; 5–30 weeks	MRI tracks Fe-labeled stem cells short-term	
*Martins-Bach * [*51*]	Metabolic profiling of *mdx* muscle	*In vitro* ^1^H NMR Spec	Lysates of *mdx* muscle; 12–24 weeks	Identified metabolites altered in *mdx* lysates	
*Xu * [*52*]	Metabolic changes in muscle after injury	^1^H NMR Spec	Injured WT & *mdx* TAs; 8 weeks	Intramuscular lipids increase post injury	Energetics deficit in necrotic phase
*Mathur * [*54*]	Effects of exercise on T2 values in muscle	T2 Mapping	*mdx* and WT; 20–60 weeks	T2, affected area up in *mdx* & after running	Affected area increased in necrotic phase
*Walter * [*33*]	Gene therapy effects on dystrophic muscle	T2 Mapping	*mdx & γsg^−/−^mice; 1 year post-therapy*	MRI tracks gene therapy efficacy in *mdx*	6 week *mdx* leg provides best stat power

*Abbreviations:* CSAmax, maximum cross-sectional area; FS, fat suppressed T2; Gd, gadolinium; GMa, gluteus maximus; GMed, gluteus medius; HSA, human serum albumin; MR, Magnetic Resonance; MG, medial gastrocnemius; PCr, phosphocreatine; RV, right ventricular; *Sg, Sarcoglycan; Sgca, Sarcoglycan alpha*; Sol, soleus; ST, semitendinosus; tATP, total adenosine triphosphate; VL, vastus lateralis; WT, wild-type.

Our power analyses and time course show the period of peak *mdx* disease provides a useful window with more severe phenotypes, ^31^P energetics phenotypes, and increased statistical power for detecting intervention effects. Here we calculate sample sizes needed to detect 20% intervention effects. In our experience with prednisone and with VBP15, we observe substantially larger than 20% intervention effects at these ages in *mdx* mice [4]. For example, fluorescent live-imaging shows a 52% decrease in markers of necrosis, and histology a 38% decrease in inflammatory foci with drug treatment [4]. Over the course of only a few weeks, we see elimination of ^31^P spectroscopy phenotypes and a dramatic reduction in the percentage of muscle affected. These findings will be valuable for design of imaging and pre-clinical therapeutic studies, by providing more phenotypes and larger differences from baseline health in controls.

Other imaging studies provide insight into *mdx* physiology (summarized in Table 2), but most avoid the critical necrotic phase of the *mdx* disease course. Cardiac MRI shows *mdx* mice can exhibit heart dysfunction by one month [45], and decreased cardiac phosphocreatine content at 8 months [29]. Agents can help visualize disrupted muscle integrity [46], [47] or detect transplanted stem cells [48]–[50]. Metabolic profiling shows alterations in injured muscle and lysates of 3- to 6-month old *mdx* mice [51], [52]. T2 mapping has been performed in 20- to 60-week-old *mdx* [53], [54]. One case study reports a single *mdx* leg assayed longitudinally to 80 weeks [55]. Dunn et al. initially showed dystrophic lesions can be detected via MRI and that crush injuries are repaired over approximately 3 weeks [53], consistent with our findings for naturally occurring *mdx* dystrophic lesions. Mathur and Vohra et al. characterized effects of exercise on *mdx*, finding effects of the *mdx* genotype and of running on muscle T2 and % affected area, with medial muscles particularly affected by running [54]. Gene correction in *mdx* and limb girdle muscular dystrophy mouse models show MRI can be used to detect therapeutic improvement in muscular dystrophy [33], [56].

The *mdx* mouse provides researchers with a genetic model of the cause of DMD (dystrophin deficiency), and MRI is emerging as an important surrogate outcome measure for muscle damage. In the present study we have found NMR phenotypes and provide new information on the dynamic disease process in *mdx* mice. Although *mdx* is typically regarded as a very mild disease model, we find ^31^P spectroscopy and T2 imaging of the 6-week old *mdx* leg show significant differences from WT mice and could provide robust outcome measures, even with relatively few animals. These findings can improve preclinical trial design by reducing the number of animals required to detect effects, allowing for longitudinal non-invasive quantification of muscle disease, and using measures that are translatable to human clinical studies.

## Supporting Information

Figure S1 **Measurement of bone sizes within hindlimb sections assayed by MRI.** Within the MRI slice stacks encompassing the 5-mm leg and 3-mm thigh regions analyzed, bone volume was assayed for each hindlimb. A) Tibia volume as measured in assayed T2 images of the leg. C) Tibial volume within the fat suppressed sections of leg that were analyzed. B) Femur volume in assayed T2 sections of the thigh. D) Femur volume as measured within assayed fat suppressed images of the thigh.
(TIF)

Table S1 **Statistical sample sizes to detect 20% intervention effects in** ***mdx*** **mice.**
(DOC)
