# Supplementary figures and images for: Non-Invasive MRI and Spectroscopy of mdx Mice Reveal Temporal Changes in Dystrophic Muscle Imaging and in Energy Deficits
Source: PLoS One. 2014 Nov 12;9(11):e112477. doi: 10.1371/journal.pone.0112477 (PMC4229202; doi:10.1371/journal.pone.0112477)

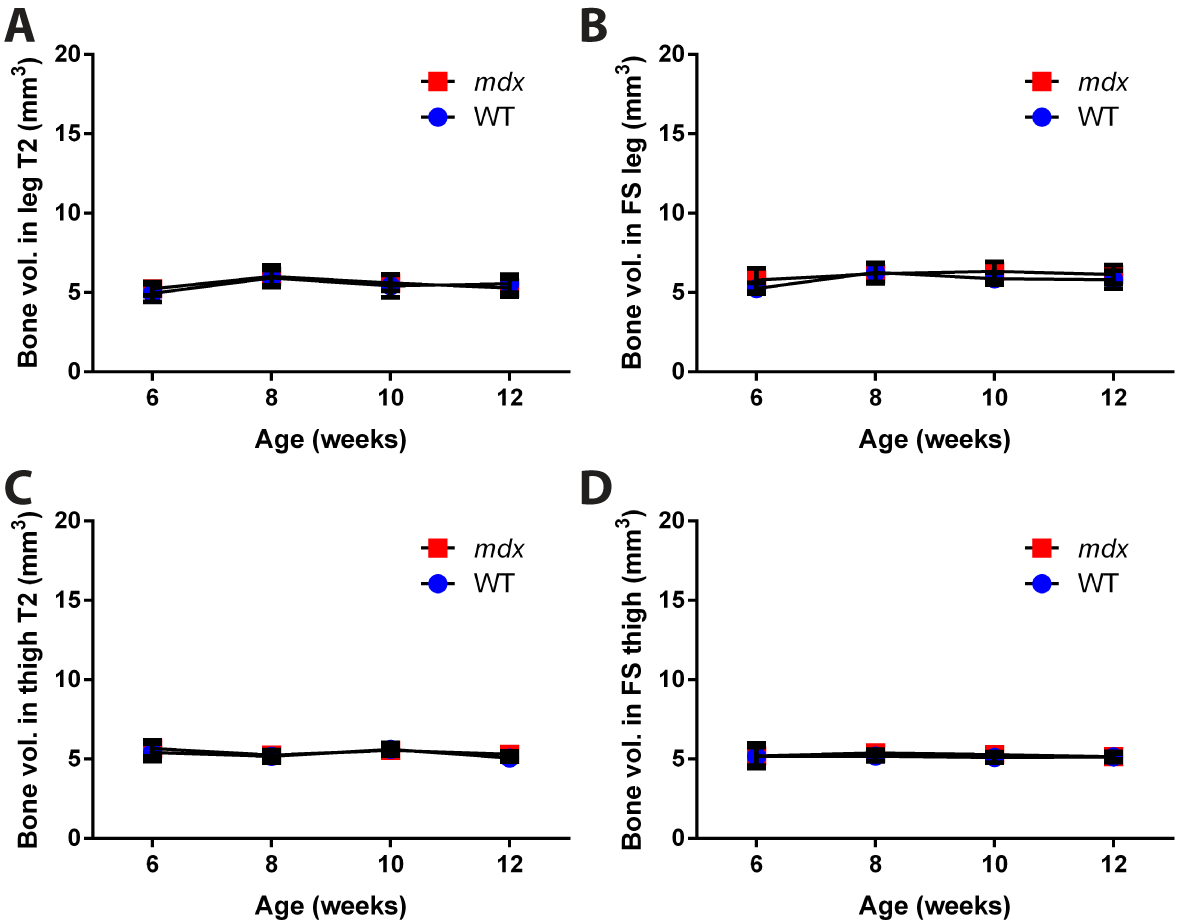

Supplement: Figure S1 — Measurement of bone sizes within hindlimb sections assayed by MRI. Within the MRI slice stacks encompassing the 5-mm leg and 3-mm thigh regions analyzed, bone volume was assayed for each hindlimb. A) Tibia volume as measured in assayed T2 images of the leg. C) Tibial volume within the fat suppressed sections of leg that were analyzed. B) Femur volume in assayed T2 sections of the thigh. D) Femur volume as measured within assayed fat suppressed images of the thigh. (TIF) [file pone.0112477.s001.tif]
