# Supplementary material for: Non-Invasive MRI and Spectroscopy of mdx Mice Reveal Temporal Changes in Dystrophic Muscle Imaging and in Energy Deficits
Source: PLoS One. 2014 Nov 12;9(11):e112477. doi: 10.1371/journal.pone.0112477 (PMC4229202; doi:10.1371/journal.pone.0112477)
Supplement: Table S1 — Statistical sample sizes to detect 20% intervention effects in mdx mice. (DOC) [file pone.0112477.s002.doc]

**Supporting Information Table 1: Statistical sample sizes to detect 20% intervention effects in *mdx* mice.**

| **Measurement type** | **Method** | **Site** | **6 weeks** | **8 weeks** | **10 weeks** | **12 weeks** |
| --- | --- | --- | --- | --- | --- | --- |
|  |  |  | **N needed**  **per group** | **N needed**  **per group** | **N needed**  **per group** | **N needed**  **per group** |
| PCr (total ATP) |  |  | 4 | N/A | N/A | N/A |
| T2 | % inflammation | Calf | 8 | 9 | 24 | 22 |
|  | Vol. inflammation | Calf | 7 | 11 | 24 | 35 |
|  | % inflammation | Thigh | 13 | 25 | 9 | 42 |
|  | Vol. inflammation | Thigh | 18 | 27 | 12 | 60 |
| Fat Suppression | % inflammation | Calf | 41 | 169 | 130 | 58 |
|  | Vol. inflammation | Calf | 39 | 166 | 121 | 65 |
|  | % inflammation | Thigh | 39 | 38 | 5 | 7 |
|  | Vol. inflammation | Thigh | 58 | 38 | 6 | 12 |
